# Exploring the Impact of Adjuvants on Vaccine Immunity Through Hematopoietic Cells

**DOI:** 10.3390/vaccines14020155

**Published:** 2026-02-05

**Authors:** Yuhyun Ji, Kavitha Bekkari, Mohammed Shardar, Geoffrey A. Walford, SamMoon Kim, Yaping Liu, Willis Read-Button, Kristina Tracy, Jennifer Kriss, Colleen Barr, Marissa Wolfle, Shailaa Kummar, Celia LaPorta, Rachel Graham, Lorenzo Chen, William James Smith, Kunal Bakshi, Nicholas Murgolo, Nicole Lea Sullivan

**Affiliations:** 1 Infectious Disease and Vaccines, Merck & Co., Inc., Rahway, NJ 07065, USA; yhji8866@gmail.com (Y.J.); nicholas.murgolo@merck.com (N.M.); 2 Translational Medicine, Merck & Co., Inc., Rahway, NJ 07065, USA; 3 Pharmacokinetics, Dynamics, Metabolism and Bioanalytics, Merck & Co., Inc., Rahway, NJ 07065, USA; 4 Data, AI & Genome Sciences, Merck & Co., Inc., Rahway, NJ 07065, USA; 5 Vaccine Drug Product Development, Merck & Co., Inc., Rahway, NJ 07065, USA; 6 Discovery Pharmaceutical Sciences, Merck & Co., Inc., Rahway, NJ 07065, USA; 7 Sterile Drug Product Commercialization, Merck & Co., Inc., Rahway, NJ 07065, USA

**Keywords:** vaccine, adjuvant, hematopoietic cells, HPV, immune outcomes

## Abstract

**Background/Objectives:** Adjuvants, added to vaccines to enhance immune responses, are central to shaping the magnitude and durability of immunity, yet their precise mechanisms remain incompletely defined. This study evaluated how diverse adjuvant combinations influence HPV vaccine immunogenicity in non-human primates, with a particular focus on impacts on hematopoietic biology—megakaryocytes and platelets—and broader innate and adaptive pathways. **Methods:** Eight adjuvanted formulations, each incorporating distinct immunomodulatory components and delivery platforms, were compared against an alum-only control in non-human primates. Longitudinal antibody titers (HPV16-specific) were measured up to 54 weeks, and blood transcriptomes were profiled at Day 1 and Day 7 after both prime and boost doses to assess pathway-level enrichment and gene-expression patterns. **Results:** Several adjuvant combinations significantly increased antibody titers at 54 weeks compared with alum alone. Formulations containing cationic lipid or monophosphoryl lipid A (MPL) were associated with enhanced antibody responses. Early upregulation of immune-related genes across innate and adaptive pathways was also observed, with some combinations (e.g., inclusion of QS21 or ISCOMs) showing similar trends. Distinct group- and time-dependent transcriptional signatures were observed, with higher-responding formulations exhibiting stronger enrichment in pathogen-influenced signaling and cellular/humoral immune programs. **Conclusions:** Adjuvant selection and formulation strategy substantially modulate vaccine immunogenicity and early transcriptional programs, including innate, adaptive, and hematopoietic pathways. While individual adjuvants differentially regulate immune and platelet-associated genes, common pathway-level patterns emerge across formulations. These findings suggest candidate mechanisms for prolonged vaccine efficacy and provide actionable insights to guide rational adjuvant design for sustained immune protection.

## 1. Introduction

Vaccination is widely recognized as one of the most powerful tools in public health for preventing infectious diseases [1,2,3]. Nonetheless, the persistence of immunity induced by vaccines can vary greatly among individuals and different vaccine types, and their underlying mechanisms are not fully understood [3,4]. One of the major challenges in the field of vaccinology is identifying the factors that contribute to immunity 24 weeks post-vaccination [1,5,6,7]. Early systems vaccinology research has shown promise in identifying molecular signatures that predict the strength of antigen-specific T cell and antibody responses [8]. However, these studies primarily focused on early indicators of peak immune responses [9]. A recent discovery has highlighted the role of megakaryocytes and platelets as unexpected contributors to sustained vaccine-induced immunity, revealing a transcriptional signature that predicts the longevity of antibody responses to vaccination and involves genes expressed in platelets and cell-adhesion pathways [1,5,10,11].

Megakaryocytes are highly specialized precursor cells that differentiate to produce blood platelets via intermediate cytoplasmic extensions known as proplatelets [10,12]. Beyond their well-recognized roles in hemostasis and thrombosis, megakaryocytes express receptors that confer immune sensing capacity, including TLR receptors [10,13]. They control the proliferation of hematopoietic cells, facilitate neutrophil egress from marrow, possess the capacity to cross-present antigen, and can promote systemic inflammation [12,14]. Megakaryocytes internalize other hematopoietic lineages, especially neutrophils, in an intriguing cell-in-cell interaction termed emperipolesis [15]. Together, these observations implicate megakaryocytes as direct participants in inflammation and immunity.

Platelets, derived from megakaryocytes, play significant roles in inflammation and immune responses [11,16,17]. Recent studies have demonstrated that megakaryocytes and platelets influence the immunological landscape through the release of extracellular vesicles and cytokines that can modulate immune cell interactions and inflammatory responses [14,16]. These extracellular vesicles are rich in various cytokines, such as IL-1, IL-6, and TNF-α, which are key mediators of the inflammatory response [11,18]. Additionally, platelets express a variety of surface molecules, including P-selectin and CD40L, which facilitate interactions with leukocytes and endothelial cells, further amplifying immune responses [18,19]. The ability of megakaryocytes and platelets to release molecules and engage in direct cell–cell interactions related to immune response underscores their potential as modulators of both innate and adaptive immunity.

In this study, we assessed the antibody levels and gene expression profiles elicited by seven different adjuvant formulations in comparison to a control group. This approach is used to evaluate the synergistic or additive effects of a new adjuvant on top of a well-established adjuvant, alum. By comparing these groups, we assess whether the additional adjuvant provides a significant improvement over alum, which is already known to be effective. The results demonstrated significant variability in immune responses, with some adjuvants, particularly alum with cationic lipid or MPL, showing notable increases in antibody production and upregulation of critical immune-related genes. These findings underscore the importance of selecting appropriate adjuvant combinations to optimize vaccine efficacy and provide valuable insights for future vaccine development strategies. The observed variations in gene expression dynamics suggest that tailored adjuvant strategies, combining multiple immunostimulatory molecules, could enhance vaccine immunogenicity, potentially leading to improved protection against infectious diseases.

We also explored signatures related to pathogen-induced pathways, cellular immune responses, and humoral immune responses. Building on this foundation, we further elucidated the patterns of megakaryocyte and platelet dynamics related to vaccine immunogenicity. By correlating RNA expression profiles with antibodies at week 54 after dose, we aimed to identify key molecular factors that can influence the longevity of immune responses elicited by different vaccine adjuvants. Given the exploratory aim of screening multiple adjuvant formulations, we adopted a breadth-focused design (4 samples per group) to compare systems-level signatures across conditions, positioning this work as hypothesis-generating rather than causal. Accordingly, these findings provide preliminary insights into the immune programs that may underlie vaccine durability and offer a rationale for the rational design of more effective vaccines that harness the immunomodulatory potential of megakaryocytes and platelets, to be validated in larger mechanistic studies.

## 2. Materials and Methods

### 2.1. Preparation of HPV VLP Vaccine with Adjuvants

The HPV vaccine was formulated using virus-like particles (VLPs) derived from the L1 major capsid protein. Recombinant L1 protein was expressed intracellularly in a *Saccharomyces cerevisiae* system [20,21,22,23]. Following cell harvesting and lysis, the self-assembled L1 VLPs were purified via chromatography. These VLPs were structurally similar to native HPV virions but lacked viral DNA. To enhance stability and immunoreactivity, the purified VLPs underwent a post-purification disassembly and reassembly process [24,25]. The final vaccine was prepared by adsorbing the VLPs onto an aluminum hydroxyphosphate sulfate (AAHS or alum) adjuvant, followed by the addition of other adjuvants as specified in Table 1.

### 2.2. Non-Human Primates Study Design

All animal studies were conducted using rhesus macaques (*Macaca mulatta*) housed at the New Iberia Research Center (NIRC) at the University of Louisiana at Lafayette. Procedures were reviewed and approved by the Institutional Animal Care and Use Committees (IACUC) of both Merck & Co., Inc. (Rahway, NJ, USA) and the University of Louisiana at Lafayette. Animal care and use throughout the study adhered to the guidelines established by the Association for Assessment and Accreditation of Laboratory Animal Care (AAALAC), the Animal Welfare Act, the American Veterinary Medical Association (AVMA) Panel on Euthanasia, and the Institute for Laboratory Animal Research (ILAR) Guide for the Care and Use of Laboratory Animals.

Prior to study initiation, each animal underwent a comprehensive health assessment conducted by a qualified veterinarian. This evaluation included a physical examination, a complete blood count, and a comprehensive chemistry panel. Additional diagnostics were performed as deemed necessary by the veterinarian to confirm the health status of the animals. Only animals confirmed to be in good health and meeting all other eligibility criteria were enrolled in the study.

All animal procedures were performed in accordance with the standards of the NIRC. Prior to vaccination and blood collection, animals were sedated with an initial intramuscular (IM) dose of ketamine hydrochloride (10 mg/kg). Sedation was maintained as needed with supplemental doses of ketamine hydrochloride (5 mg/kg). The vaccine was administered as two 0.5 mL IM injections, one into each quadriceps muscle, for a total dose of 1.0 mL per animal. To avoid interference with the vaccination site, all sedation agents were administered at an alternate anatomical location.

**Figure 1:**
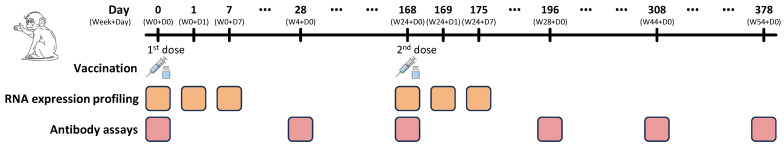
Study Design for Evaluating HPV Vaccine Immunogenicity with Diverse Adjuvants. The experimental design for assessing the immunogenicity of the HPV vaccine across eight groups, each consisting of four monkeys. The study involves two vaccine doses administered 24 weeks apart. RNA samples were collected at day 0 (pre-dose baseline), day 1, and day 7 following both the prime and boost doses. Antibody responses specific to HPV type 16 were measured at weeks 0 (pre-dose baseline), 4, 24, 28, 44, and 54 to evaluate short-term response and long-term vaccine immunogenicity. This comprehensive approach aims to elucidate the effects of various adjuvants on the immune response over time.

Non-human primates (NHPs) were assigned to treatment groups (n = 4 per group) to receive different adjuvanted vaccine formulations as detailed in Table 1. The vaccination regimen consisted of a two-dose series administered intramuscularly. The initial dose (Day 0) was followed by a second dose at Week 24. For each administration, a total volume of 1.0 mL was delivered as two separate 0.5 mL injections, one into each quadriceps muscle. The overall study design is depicted in Figure 1.

### 2.3. Immune Profiling—RNA Sequencing

To evaluate the effect of different adjuvants on immune gene expression, peripheral blood mononuclear cells (PBMCs) were analyzed. Blood samples were collected at six time points: pre-dose (baseline), and at 1 and 7 days following each of the two vaccinations, as depicted in Figure 1. Whole blood was preserved in PAXgene Blood RNA Tubes. Total RNA was subsequently isolated using the PAXgene Blood RNA Kit (PreAnalytix, Hombrechtikon, Switzerland) following the manufacturer’s protocol. The quality and quantity of the isolated RNA were assessed using an Agilent Bioanalyzer with an RNA 6000 Nano LabChip (Agilent Technologies, Santa Clara, CA, USA).

For samples collected in PAXgene tubes, cells were pelleted by centrifugation at 10,000× *g* for 6 min. The supernatant was discarded, and the cell pellet was washed by resuspension in 1 mL of dimethylpyrocarbonate (DMPC)-treated water, followed by a second centrifugation step at 10,000× *g* for 6 min. For samples in all other media, cells were pelleted by centrifugation at 2000× *g* for 30 min. After removing the supernatant, all cell pellets were disrupted by vortexing in the appropriate lysis buffer for the subsequent extraction method.

Total RNA was extracted from samples using standard methods designed to yield high-integrity RNA. RNA sequencing libraries were then constructed using the TruSeq Stranded Total RNA Ribo-Zero kit (Illumina, San Diego, CA, USA) following the manufacturer’s protocol. This procedure depletes ribosomal RNA (rRNA) to enrich for both coding and non-coding transcripts while preserving strand information. The prepared libraries were sequenced on an Illumina platform, generating paired-end 50-base-pair reads. Sequencing depth and multiplexing strategy were optimized based on the experimental design to achieve adequate coverage for transcriptomic analysis. Gene orthologs were identified by cross-referencing gene IDs with the Ensembl BioMart database.

### 2.4. Multiplexed Meso Scale Discovery (MSD) Assay

**Figure 2:**
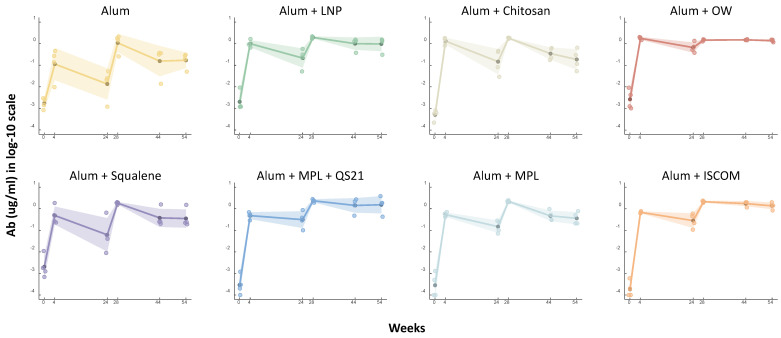
Longitudinal Antibody Levels Against HPV Type 16 in Response to Adjuvants. Antibody levels against HPV type 16 across different adjuvant groups, including the control group, alum only. Data points of each animal (four points per group) at four different time points are shown with colored dots. The darker dots connecting thick solid lines represent the geometric means of antibody levels, while the shaded areas indicate the geometric standard deviation. This analysis highlights the efficacy of the adjuvant combinations in boosting immune responses over time.

To assess the immunogenicity of the adjuvanted vaccines, serum antibody binding to HPV VLPs was quantified using a multiplexed Meso Scale Discovery (MSD) electrochemiluminescence assay (Figure 2). Custom 96-well MSD plates, pre-coated with 90 µg/mL of HPV type 16 VLPs, were used for the analysis. Prior to use, plates were equilibrated to room temperature (RT). Each well was then blocked for 30 min at RT with 150 µL of 3% nonfat milk in PBST (PBS with 0.05% Tween 20) while shaking at 400 rpm. Following blocking, the plates were washed three times with 300 µL of PBST using an automated plate washer (BioTek, Seattle, WA, USA).

NHP serum samples were diluted 1:100 in an assay buffer consisting of PBST with 1% fetal bovine serum (FBS) using an Agilent BRAVO automated liquid handler (Agilent Technologies, Santa Clara, CA, USA). To quantify antibody concentrations, a reference standard was prepared by pooling known concentrations of HPV-specific mouse monoclonal antibodies (mAbs) corresponding to each of the nine HPV types. This reference standard cocktail was then used to generate a 7-point standard curve through a 5-fold serial dilution in the assay buffer. The assay buffer alone served as the blank control for the assay.

Diluted NHP serum samples and the reference standards were added to the prepared MSD plates at 50 µL per well. The plates were then incubated for 1 h at room temperature (RT) with shaking (400 rpm). Following this incubation, the plates were washed three times with 300 µL of PBST. Next, a SULFO-TAG labeled detection antibody was added at 50 µL per well. For wells containing the standard curve, goat anti-mouse IgG was used, while goat anti-NHP IgG was used for wells with NHP sera; both were diluted to 0.5 µg/mL in assay buffer. The plates were incubated for another hour at RT with shaking (400 rpm) and then washed three final times with 300 µL of PBST. Finally, 150 µL of MSD Read Buffer was added to each well, and the plate was analyzed on an MSD Meso Sector S600 instrument (Meso Scale Discovery, Rockville, MD, USA). HPV type-specific antibody concentrations in the unknown samples were calculated by fitting the standard curves using a 4-parameter logistic regression model.

### 2.5. Data Preparation for Computational Analysis

**Figure 3:**
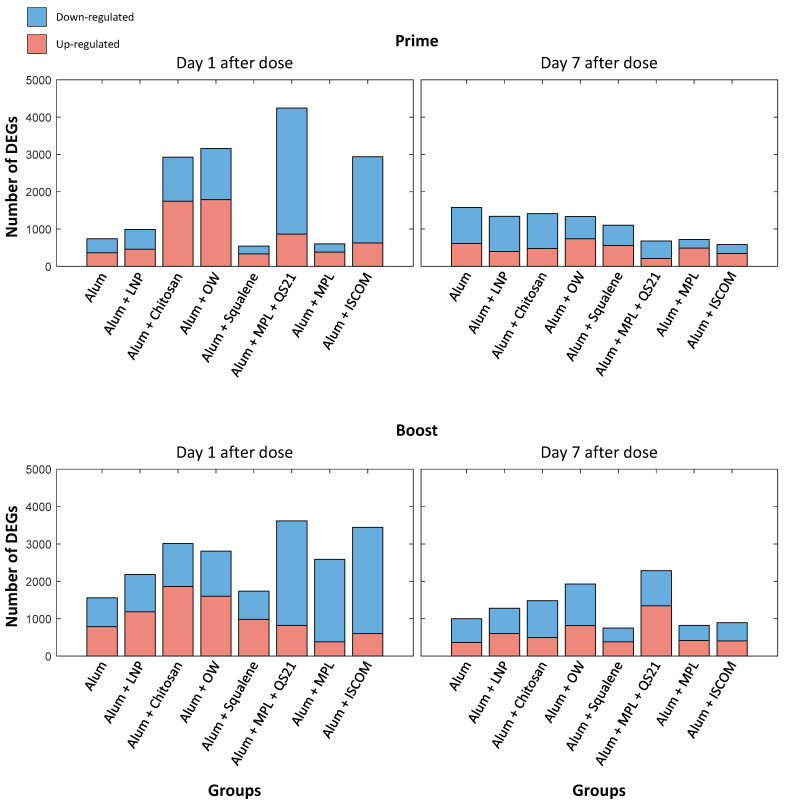
Differentially Expressed Genes Post-Vaccination. Comparisons of the differentially expressed genes (DEGs) identified on day 1 and day 7 following the prime and boost doses of the HPV vaccine. Upregulated genes are indicated in red, while downregulated genes are shown in blue. The number of DEGs is presented with a stacked bar plot, with significance defined as *p* < 0.05 and an absolute value of log2 fold change > 0.2. A two-sided paired *t*-test was employed to compute *p* values for each group, highlighting the impact of adjuvants on gene expression dynamics post-vaccination.

**Figure 4:**
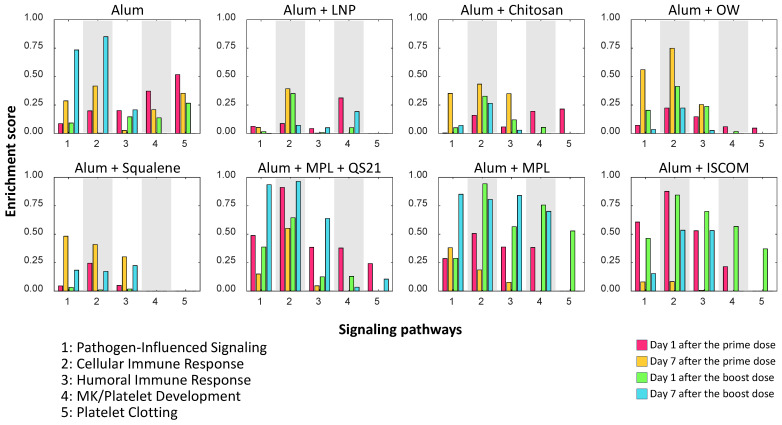
Enrichment Scores of Differentially Expressed Genes. Illustration of the expressed gene levels related to pathogen-induced signaling, cellular immune response, humoral immune response, megakaryocyte/platelet development, and platelet clotting. The enrichment score quantifies the overrepresentation of DEGs within specific biological pathways or processes across four time points (day 1 and day 7 following both the prime and boost doses) and eight experimental groups. The enrichment scores for each gene group are displayed to visualize the DEG levels post-vaccination, providing insights into the biological relevance and functional impact of the gene expression changes. The statistical significance of the enrichment was assessed using the hypergeometric distribution. A higher enrichment score suggests a significant enrichment of DEGs in the gene set, highlighting the biological relevance of the observed gene expression changes.

**Figure 5:**
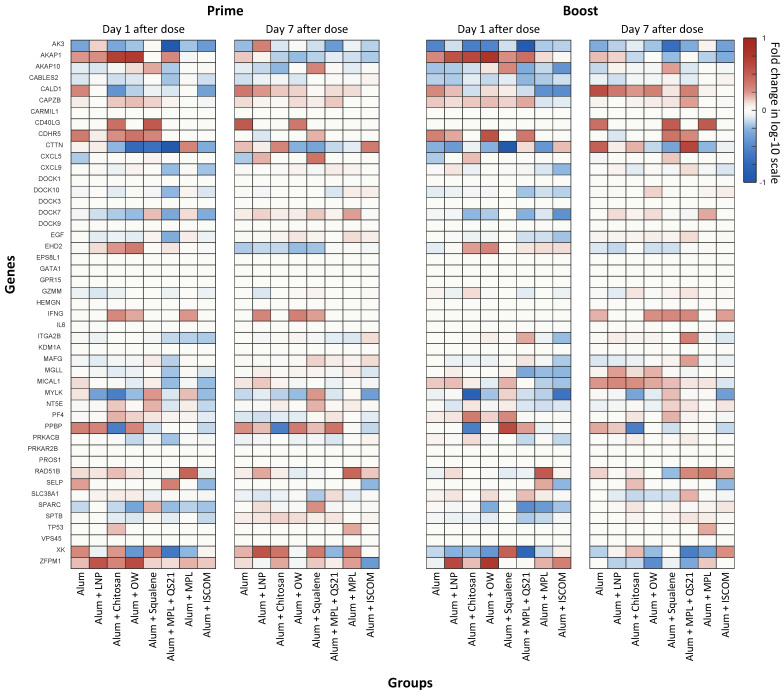
Heatmap of Gene Expression Levels Related to Megakaryocyte/Platelet Development Across Adjuvant Groups. The heatmap depicts the expression levels of genes related to megakaryocyte/platelet development across different adjuvant groups. Each row represents an individual gene expression level, while each column corresponds to a specific adjuvant group. The color gradient indicates the relative expression levels, allowing for a visual comparison of how various adjuvants modulate gene expression in distinct ways. This approach facilitates a clearer understanding of how specific genes respond to adjuvanted vaccination, aiding in the identification of potential targets for further investigation.

**Figure 6:**
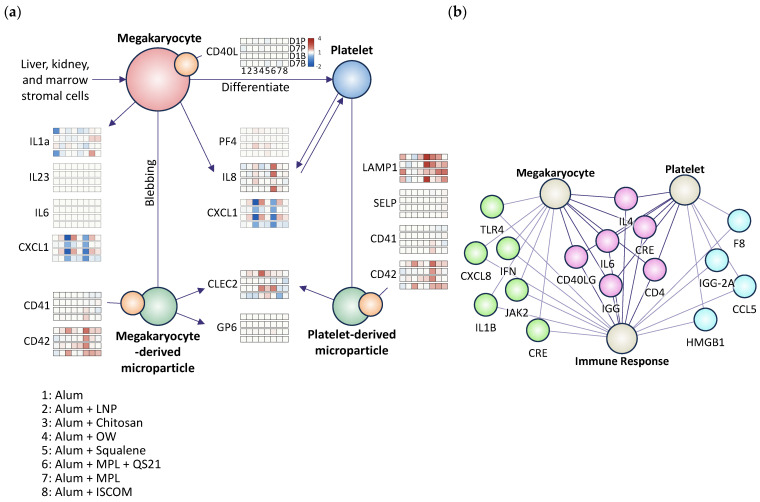
Gene Ontology Analysis from Curated Signaling Pathways and Generative AI. Presentation of a comparative analysis of Gene Ontology (GO) terms derived from the Ingenuity Pathway Analysis (IPA) and BenchSci Ascend database, alongside signaling pathways curated from a comprehensive literature review. (**a**) The signaling pathways were curated from the previously studied literature reviews. The arrows indicate the relationships between terms and the direction of the biological processes. The red dot means the megakaryocyte (MK). Blue dot means a platelet. The green dot means microparticles. The orange dot means the membrane components on each cell/particle. Each arrow connects the related proteins or cytokines. The gene expression levels are shown on the right side of the label with the 4 by 8 heatmap, where each row represents the time points (day 1 and day 7 after the prime and boost dose) and each column represents the groups from 1 to 8. (**b**) Network diagram illustrating the interconnections among megakaryocytes, platelets, and the immune response, generated by AI based on previously studied research. This platform is to streamline the discovery of relevant biological connections and enhance research efficiency by providing access to a comprehensive database of scientific literature. The connecting lines indicate that they are related or that they may be related. The magenta dots represent molecules that have been commonly and frequently studied in relation to megakaryocytes, platelets, and the immune response. The lime dots indicate molecules that have established relationships with both megakaryocytes and the immune response, while the cyan dots denote molecules associated with platelets and the immune response. The analysis highlights the overlap and discrepancies between the automated GO annotations and the manually curated pathways, providing insights into the biological processes and molecular functions relevant to the study. This comparison aims to identify potential gaps in current knowledge and suggest directions for future research, emphasizing the importance of integrating computational and manual approaches in understanding complex signaling networks.

RNA sequencing data were normalized to enable comparative gene expression analysis. The fragments per kilobase of transcript per million mapped reads (FPKM) values were log10-transformed using the formula log10(FPKM + 0.01). A pseudo-count of 0.01 was added to prevent errors from zero values and to symmetrize the representation of up- and down-regulated genes. To remove low-quality data, transcripts with a transformed value below −1 were filtered out. This filtering step reduced the dataset from 35,398 to 12,985 transcripts. The gene IDs for the remaining data were converted to their human orthologs using the Ensembl BioMart database. For subsequent analysis, a curated list of immune-, megakaryocyte-, and platelet-related genes was selected. The expression patterns of representative genes from this list are illustrated in Figure 3, Figure 4, Figure 5 and Figure 6.

### 2.6. Enrichment Scores for Analyzing Differentially Expressed Genes

To assess the biological significance of differentially expressed genes (DEGs) associated with signaling pathways, including platelet aggregation and clotting, enrichment analysis using the hypergeometric test was performed. First, a gene set comprising genes known to be involved in pathogen-influenced signaling, cellular immune response, humoral immune response, megakaryocyte/platelet development, and platelet clotting, sourced from relevant databases, including the Ingenuity Pathway Analysis (IPA) program, BenchSci ASCEND, and literature studies, was defined as shown in Figure 4. Second, genes with a *p*-value < 0.05 and an absolute value of log2 fold change > 0.2 relative to baseline (pre-dose) were considered significantly changed in expression to filter up- and down-regulated genes. To be specific, fold change was defined as the ratio of post-dose to baseline, computed from log10-transformed values by taking their difference. For statistical testing, we applied a two-sample *t*-test across animals to obtain a nominal *p*-value per gene for the baseline versus post-dose comparison. Genes were classified as differentially expressed only if they met both an effect-size and a significance criterion: the log10 fold change exceeded 0.2 in either direction and the corresponding *p*-value was below 0.05. This combination of a modest effect-size threshold and a nominal significance cutoff was selected a priori to balance sensitivity and specificity in an exploratory, small-sample design (n = 4 per group). It avoids flagging trivially small shifts while remaining permissive for biologically plausible changes under adjuvant stimulation. Given the limited power for exhaustive single-gene discovery, we treat gene-level findings as associative and place emphasis on pathway-level patterns in the Results. Third, to assess whether a given set of genes was statistically overrepresented among the differentially expressed genes, enrichment analysis was performed using the hypergeometric cumulative distribution. The hypergeometric cumulative distribution evaluates whether specific gene sets were statistically overrepresented. For each gene set, the hypergeometric cumulative distribution estimates the probability (enrichment score) of observing a given number of overlapping genes between our list of expressed genes with significant change and a predefined gene set, such as pathogen-influenced signaling and megakaryocyte/platelet development. Specifically, the MATLAB (The MathWorks Inc. (2021), Natick, MA, USA, R2021a) function “hygecdf” was used for this statistical analysis, with input values corresponding to: the total number of genes analyzed, the number of genes in each pathway gene set, the number of statistically significant DEGs, and the number of overlapping genes between the significant DEGs and each pathway gene set.

## 3. Results

### 3.1. Non-Human Primates Study with 8 Different Adjuvants for the HPV Vaccine

The 8 adjuvants were utilized (Table 1), including alum as a mineral salt adjuvant. The first group, which has already been used for the HPV vaccine (HPV + Alum), served as a control group. The adjuvants evaluated were differentiated by their core immunostimulatory components and their formulation technology. The delivery platforms included various systems, such as liposomes and lipid nanoparticles, each designed to potentiate the immune response in a distinct manner. This combination aims to demonstrate enhanced immunogenicity of the vaccines, likely due to the activation of additional immune stimulatory pathways that facilitate better antigen presentation and influence memory cells.

To investigate the immunogenicity of vaccine responses elicited by various adjuvant types, an experiment measuring antibody levels was conducted, as shown in Figure 1, and the result is shown in Figure 2. Figure 2 illustrates the longitudinal antibody levels against HPV type 16 in non-human primates receiving different adjuvant formulations, including the alum-only control group. This experimental design also enables monitoring of how different adjuvants modulate immune responses through RNA expression analysis of the HPV vaccine. The database for differentially expressed RNA gene analysis was derived from the Ingenuity Pathway Analysis (IPA) program, BenchSci ASCEND, and literature studies (Methods). For a more focused analysis, we selected specific gene sets associated with key immunological processes. These included genes related to the cellular immune response, the humoral immune response, as well as megakaryocyte and platelet biology. The expression patterns of these selected genes are presented in Figure 3, Figure 4 and Figure 5.

### 3.2. Longitudinal Antibody Profile of Adjuvanted Vaccines

The antibody levels against HPV type 16 were evaluated at multiple time points following vaccination with various adjuvanted formulations. The study included a control group receiving the HPV vaccine with alum adjuvant and eight experimental groups, each incorporating different immunostimulatory molecules alongside alum (Table 1). Antibody levels were measured at day 0 (pre-dose) and week 4 post-prime and boost doses, and at weeks 44 and 54 to assess both the immediate and long-term immune responses (Figure 1 and Figure 2). The results demonstrated a significant increase in antibody levels at weeks 4 and 54 for all groups compared to the control group (Group 1, HPV + alum only), as shown in Figure 2. For each group, the geometric mean antibody titers are shown as thick solid lines, with shaded regions representing the geometric standard deviation, indicating variability within each group (Figure 2). Comparison across groups reveals that several adjuvant combinations elicited higher and more sustained antibody responses compared to the alum-only control.

Notably, Group 6 (Alum + MPL + QS21), which received alum combined with MPL and QS21 in a liposomal formulation, exhibited the highest antibody response, achieving a remarkable 14.42-fold increase compared to the control group at week 54. This substantial enhancement suggests that the combination of MPL and QS21 effectively promotes both humoral and cellular immunity, indicating the potential of this formulation to generate a robust antibody response. Similarly, Group 7 (Alum + MPL), which received alum combined with MPL in an alum-absorbed suspension, demonstrated a 9.33-fold increase in antibody levels. It is known that the addition of MPL can create a stronger and longer-lasting immune response compared to the vaccine formulated with just aluminum alone [2]. Considering that Groups 6 and 7 both contain the MPL along with the extra amount of alum, the results suggest that MPL with the increased alum-absorbed suspension allows for more prolonged immune stimulation and higher antibody titers in the case of the HPV vaccine for NHPs.

In addition, Group 6 (Alum + MPL + QS21) exhibited the highest antibody response, aligning with early transcriptional enrichment across innate and MK/platelet pathways observed in this group. Similarly, Group 8 (Alum + ISCOM) showed a high increase in antibody response and early platelet-associated pathway enrichment, further supporting the alignment between MK/platelet activity and antibody magnitude. Group 7 (Alum + MPL) demonstrated a slight increase in antibody levels, concurrent with early activation of MK/platelet-linked signatures and high humoral immune response after the boost dose. Overall, across groups, those with more pronounced MK/platelet transcriptional activity tended to exhibit higher downstream antibody titers, reinforcing an associative link between early platelet-associated programs and humoral immunity (Figure 4).

Group 4 (Alum + OW), which contains alum combined with cationic lipid in an oil-in-water emulsion, resulted in an 8.13-fold increase in antibody levels at week 54. The use of cationic lipids in this formulation might have facilitated antigen presentation and uptake by immune cells, contributing to the enhanced antibody production observed [2,3]. Additionally, Group 8 (Alum + ISCOM), which received alum combined with an immune-stimulating complex (ISCOM) in a cage-like nanoparticle formulation, showed a 7.21-fold increase in antibody response. ISCOMs are known for their ability to enhance the immunogenicity of antigens by promoting their delivery to antigen-presenting cells, thereby boosting the overall immune response [2,3].

These findings suggest that combining different types of immunostimulatory molecules into vaccine formulations not only increases vaccine immunogenicity against the HPV vaccine but also highlights the potential to enhance the antibody response of adjuvanted vaccines. The results underscore the importance of combining adjuvants to stimulate multiple signaling pathways to maximize vaccine immune responses.

### 3.3. Analysis of Differentially Expressed Genes Using Bulk RNA Sequencing Data

To investigate the immune response elicited by the various adjuvants, RNA sequencing was performed on blood samples collected at two intervals: 1 day and 7 days post-prime and boost doses, as shown in Figure 1. The analysis was to focus on identifying DEGs at these time points to understand the temporal dynamics of the immune response (Figure 3, Figure 4, Figure 5 and Figure 6).

Both prime and boost doses significantly upregulated and downregulated genes at 1 day post-dose (Figure 3) compared to the pre-dose, which was used as the baseline. Genes with a *p*-value < 0.05 and an absolute value of log2 fold change > 0.2 were considered significantly differentially expressed when plotting Figure 3. The gene expression changes at 1 day post-dose were more pronounced compared to those at 7 days post-dose (Figure 3). In Group 1 (Alum), which served as the control with alum only, a relatively lower number of DEGs were observed on day 1 following the prime dose compared to Groups 3 (Alum + Chitosan), 4 (Alum + OW), 6 (Alum + MPL + QS21), and 8 (Alum + ISCOM). However, Group 1 (Alum) showed a higher number of DEGs compared to the other groups on day 7 after the prime dose. Group 1 (Alum) exhibited a modest initial increase in antibody level, while the antibody level showed a relatively lower antibody response than other groups at later time points in NHPs (Figure 2). The minimal gene expression changes of Group 1 (Alum) at day 1 and day 7 after the boost dose, especially in immune-related pathways shown in Figure 4, likely correlate with the less durable antibody response (Figure 2, Figure 3 and Figure 4) in NHPs.

Group 6 (Alum + MPL + QS21) exhibited the most substantial number of DEGs, particularly on day 1 post-prime dose, with pronounced upregulation of genes associated with immune responses (Figure 3). Likewise, Group 6 showed the most significantly increased antibody level at week 54 compared to the control group, alum only, by 14.42-fold (Figure 2). Group 4 (Alum + OW), which increased antibody response more than 8 times that of the control group, demonstrated a moderate number of DEGs with balanced expression of upregulated and downregulated genes, notably involving immune response pathways. These gene expression changes correlated with a significant and sustained increase in antibody levels, suggesting enhanced vaccine immunogenicity. Similarly, Group 8 (Alum + ISCOM), which showed increased antibody response, also showed a high number of DEGs, indicating active modulation of gene expression resulted in the increased vaccine immunogenicity.

### 3.4. Enrichment Scores of Differentially Expressed Genes

The enrichment scores for each gene group are displayed in Figure 4 to visualize the DEG levels of different pathways after vaccination, providing insights into the biological relevance and functional impact of the gene expression changes (Methods). The enrichment analysis focused on the following categories: Pathogen-influenced signaling, cellular immune response, humoral immune response, megakaryocyte/platelet development, and platelet clotting (Figure 4).

Early after vaccination (day 1 post-prime and post-boost), DEGs were enriched in pathogen-influenced signaling and cellular immune response pathways generally across the groups, indicating rapid activation of innate immunity. By day 7, enrichment shifted toward humoral immune response pathways, especially after the boost shot. Importantly, megakaryocyte/platelet development and platelet clotting pathways were not commonly differentiated across multiple adjuvant groups and time points.

Particularly, Group 6 (Alum + MPL + QS21) and Group 8 (Alum + ISCOM) showed significantly high cellular immune response at day 1 after the prime. Also, Group 6 (Alum + MPL + QS21) and Group 1 (Alum) showed significantly high cellular immune response at day 7 after the boost. Notably, Group 6 showed the highest score in cellular immune response and pathogen-influenced signaling at day 7 after the boost dose. Considering that Group 6 (Alum + MPL + QS21) exhibited the highest vaccine immunogenicity at week 54, it is plausible that the cellular immune response and pathogen-influenced signaling pathways play a significant role in sustaining vaccine immunogenicity, which is consistent with previously reported findings [2].

However, the second-highest scores of cellular immune response at day 7 after the boost dose were observed in Group 1 (Alum). Also, Group 1 (Alum) has shown a relatively high score of pathogen-influenced signaling at day 7 after the boost dose. Although some signals in Group 1 (Alum) were relatively high compared to others, again, the antibody levels at weeks 44 and 54 were the lowest, highlighting the need for further research into how immune signals correlate with vaccine immunogenicity.

In Figure 4, the enrichment analysis of the megakaryocyte/platelet development pathway reveals distinct group- and time-dependent patterns following HPV vaccination with various adjuvants. We observed that Group 1 (Alum) and Group 6 (Alum + MPL + QS21) exhibited pronounced activation at day 1 after the prime dose. On the other hand, Group 7 (Alum + MPL) and Group 8 (Alum + ISCOM) showed high enrichment scores at day 1 after the boost dose. On day 1 post-dose, Group 2 (Alum + LNP) exhibited relatively high MK/platelet enrichment compared with its other pathway signals. Its MK/platelet score remained similar to those observed in the other adjuvant groups that showed high antibody response. This temporal trend indicates that the megakaryocyte/platelet development pathway tends to show high activation at day 1 after dose, suggesting robust engagement of megakaryocytes/platelets in an early stage of this immunological system. Although all the Groups contain alum as an immunostimulatory molecule, their effects on the megakaryocyte/platelet-related pathway were varied.

Across adjuvant groups, platelet clotting pathway enrichment (Figure 4, panel 5) displayed clear time- and adjuvant-dependent variation. Early after the prime dose, Groups 1 (Alum) and 6 (Alum + MPL + QS21) showed stronger clotting signatures, which coincided with heightened pathogen-influenced and cellular immune pathways in the same window. Following the boost, Groups 7 (Alum + MPL) and 8 (Alum + ISCOM) exhibited higher clotting enrichment that aligned temporally with increased humoral pathway scores. Group 3 (Alum + Chitosan) and 6 (Alum + MPL + QS21) demonstrated relatively elevated clotting signals compared with other groups at day 1. Taken together, the platelet clotting program tracks with broader innate and humoral activation in an adjuvant- and time-specific manner. However, the clotting pathway–related genes were not sufficiently differentiated from baseline and showed limited differences across groups and time points. This constraint limits interpretability and makes it difficult to draw stronger connections between clotting signatures and other immune pathways.

### 3.5. Heatmap of Differentially Expressed Genes Related to Megakaryocytes and Platelets

Given our focus on megakaryocytes and platelets, an in-depth analysis of genes related to megakaryocyte/platelet development was conducted using a heatmap. The heatmap in Figure 5 and Figure 6a is generated to visualize the expression patterns of these genes across different time points and adjuvant groups. Specifically, Figure 5 and Figure 6a illustrate the expression levels of megakaryocyte/platelet development-related genes at 1 day and 7 days post-prime and boost doses, providing insights into their potential roles in promoting effective hemostasis and immune activation.

Several genes exhibited consistent expression patterns across both time points and vaccine doses. For instance, *AK3* (Adenylate kinase 3, which plays a role in cellular energy metabolism, including in megakaryocytes) and *CABLES2* (Cdk5 and Abl enzyme substrate 2, involved in regulating the cell cycle and apoptosis) were generally downregulated at all measured intervals. In contrast, *CAPZB* (a key regulator of actin filament dynamics, essential for proper platelet function and formation) consistently showed upregulation across both time points and doses [37,38,39]. The uniformity of these expression trends suggests that these genes may play fundamental roles in mediating vaccine-induced immunity and could represent important targets for further investigation.

In contrast, other genes displayed time-dependent expression changes. For instance, *AKAP1* (A-kinase anchoring protein 1, which localizes protein kinase A to specific cellular compartments and influences platelet signaling pathways) and *EHD2* (Eps15 homology domain-containing 2, involved in endocytosis and membrane trafficking) were upregulated at day 1 post-dose but downregulated by day 7, indicating their involvement in the early phase of the immune response [40,41]. Conversely, *SPARC* (secreted protein acidic and rich in cysteine, which mediates cell-matrix interactions and modulates platelet function during clot formation and tissue repair) was downregulated at day 1 but upregulated at day 7, suggesting a potential role in the later stage of the immune response [42].

Particularly, Groups 6 (Alum + MPL + QS21) and 8 (Alum + ISCOM), which exhibited high immunogenicity for the HPV vaccine, generally downregulated genes related to megakaryocytes and platelets at day 1 after the prime dose. While Group 4 (Alum + OW), which exhibited the second highest immunogenicity in antibody level, showed a mixed pattern of gene expression level on 1 day after the prime dose. Above all, Group 5 (Alum + Squalene) consistently upregulated *AKAP10* (A-kinase anchoring protein 10: regulating signaling pathways that affecting platelet activation), *MYLK* (mediates phosphorylation for platelet shape change and spreading), and *NT5E* (inhibits platelet activation and aggregation, regulating clot formation and modulating vascular inflammation) across both time points and doses, indicating its potential importance in the immune response related to megakaryocytes and platelets [40,43,44].

### 3.6. Generative AI for Connecting Immune Response and Megakaryocytes/Platelets

To understand the existing knowledge and construct a navigation map illustrating the relationship between immune responses and platelet/megakaryocyte biology, the ASCEND platform from BenchSci was utilized [45]. ASCEND is a generative AI platform designed for exploring biological data and their interactions [45]. It visualizes complex relationships and integrates literature with experimental findings derived from closed-access publishers and open-access journal articles, including Springer Nature, Wiley, and bioRxiv. This platform aggregates and analyzes data from various scientific publications, providing valuable insights into the interactions among different biological entities.

Using ASCEND, key components of the immune response (e.g., cytokines, immune cells) and factors related to platelets and megakaryocytes (e.g., growth factors, signaling pathways) were identified based on a comprehensive literature review. The resulting Figure 6b illustrates the cytokines, genes, and pathways associated with platelet activation, linking megakaryocytes/platelets to immune cell activation.

The map highlights several molecules, such as IL-4, IL-6, and CD4, which are known to influence platelet activation and megakaryocyte differentiation [46]. These cytokines have been shown to enhance platelet production under inflammatory conditions [46]. Additionally, the interactions between various immune cells (e.g., macrophages, T cells) and megakaryocytes are depicted, demonstrating how signals derived from immune cells can modulate megakaryocyte activity and platelet release.

## 4. Discussion

In this study, we evaluated the antibody levels elicited by seven different adjuvant formulations in comparison to a control group (alum). The results demonstrated a range of antibody responses across the various adjuvant groups, highlighting the differential immunogenicity of each formulation in enhancing immune responses. The control group was serving as a baseline for comparison. Among the adjuvanted groups, the antibody levels varied, with some formulations showing notable increases in antibody response. Specifically, Groups 4 (Alum + OW), Group 6 (Alum + MPL + QS21), and Group 8 (Alum + ISCOM) indicated a positive enhancement in immune response compared to the control, suggesting that these adjuvants may effectively stimulate antibody production in NHPs. Several adjuvant groups (Groups 2 (Alum + LNP), 3 (Alum + Chitosan), 5 (Alum + Squalene), and 7 (Alum + MPL)) displayed an increase that was similar to, or slightly higher than, that of the control group, indicating that not all adjuvant combinations were equally effective in promoting an immune response. The variability observed underscores the importance of selecting appropriate adjuvant formulations to optimize vaccine immunogenicity.

All experimental groups that included immunostimulatory molecules beyond alum exhibited increased antibody levels and enhanced vaccine immunogenicity. This demonstrates that the combined use of adjuvants and the stimulation of various immune pathways can significantly improve vaccine immunogenicity. Overall, these findings deepen our understanding of how different adjuvants can modulate antibody responses, offering valuable insights for future vaccine development strategies. The observed variations in gene expression dynamics suggest that tailored adjuvant strategies by combining multiple chemicals could enhance the immunogenicity of vaccines, potentially leading to improved protection against infectious diseases. However, it is already well established that the HPV vaccine Gardasil^®^9 using alum demonstrated excellent efficacy in humans. The discrepancy between our experimental results and these findings may be due to the fact that our study was conducted in NHPs. Since a robust foundation for directly comparing the immune systems of humans and monkeys has not yet been established, additional experiments may be necessary.

We also investigated the effects of various adjuvant formulations on the immune response elicited by the HPV vaccine, focusing on differentially expressed genes and their associated biological processes. The results demonstrated significant variations in gene expression profiles across the eight adjuvant groups, highlighting the differential efficacy of each formulation in enhancing immune responses. The analysis revealed that certain adjuvants, particularly those represented by Groups 3 (Alum + Chitosan), 4 (Alum + OW), 6 (Alum + MPL + QS21), and 8 (Alum + ISCOM), led to a notable increase in upregulated genes associated with critical immune functions. Specifically, Group 6 (Alum + MPL + QS21) has shown significantly increased pathogen-influenced signaling and cellular immune response gene expression level at day 7 after the boost shot. The robust gene expression changes are likely linked to the strong and durable antibody response observed in this group, highlighting the impact of adjuvant-induced gene modulation on vaccine efficacy. We observed that adjuvant groups with stronger early enrichment in MK/platelet pathways often exhibited high innate and humoral signaling. While these relationships are correlative and derived from a small cohort, the temporal concordance suggests that platelet-linked transcriptional activity may serve as an early systems-level marker that tracks with the magnitude of humoral responses across adjuvants.

Our systems-level analyses reveal adjuvant-dependent differences in early innate, humoral, and megakaryocyte/platelet-associated signatures that align with subsequent antibody magnitude. Consistent with the antibody kinetics in Figure 2, Groups 6–8 showed higher enrichment across pathogen-influenced signaling, humoral and cellular immune programs, MK/platelet development, and platelet clotting (Figure 4), supporting an associative concordance between early pathway activation, MK/platelet signaling, and later antibody responses across these formulations. Although these patterns are robust across time points and internally consistent with the observed antibody outcomes, we interpret them as hypothesis-generating rather than causal, given the small sample size and exploratory design. Consequently, these findings suggest that early MK/platelet-associated programs may serve as useful systems-level indicators for prioritizing adjuvant formulations—a premise that warrants validation in larger cohorts with cell-resolved profiling and targeted perturbation studies to establish the mechanism.

The significant changes in gene expression, particularly in pathways related to immune activation, correlated with the observed robust and sustained antibody levels, underscoring the role of gene expression dynamics in supporting vaccine immunogenicity. The consistent and significant changes in expression observed across multiple groups and time points underscore the complex interplay between these genes and the immune response. These variations in gene expression levels can provide insights into the mechanisms by which adjuvants affect platelet development and function, potentially identifying key genes that could serve as targets for further investigation. These findings may guide future research on vaccine durability and efficacy, potentially identifying biomarkers that can predict long-lasting immunity. Understanding the molecular mechanisms underlying these gene expression patterns could pave the way for the rational design of more effective vaccines that harness the immunomodulatory potential of megakaryocytes and platelets.

## 5. Conclusions

In summary, this non-human primate study demonstrates that adjuvant formulation profoundly shapes the magnitude and durability of HPV16-specific antibody responses, with several combinations—particularly Group 6 (Alum + MPL + QS21), Group 8 (Alum + ISCOM), and Group 4 (Alum + OW)—eliciting higher and more sustained titers than alum alone. These immunogenicity patterns are mirrored by distinct transcriptional programs over early time points, including stronger enrichment in pathogen-influenced signaling and cellular/humoral immune pathways in the higher-responding groups. Together, the antibody kinetics and gene-expression profiles indicate that leveraging complementary immunostimulatory components and delivery platforms can enhance vaccine performance by engaging broader immune circuitry. While alum-based HPV vaccines are highly efficacious in humans, our NHP results underscore formulation-dependent differences and highlight the value of systems-level readouts to inform adjuvant selection. These findings provide a rationale for prioritizing multi-component adjuvant strategies and motivate future studies that extend cohort size, incorporate cell-resolved profiling, and refine formulation parameters to optimize durability and translate insights across species.

## Figures and Tables

**Table 1:** Adjuvants Used for Non-human Primate Study and Their Formulation Strategies. Various adjuvant types and formulation strategies utilized in the non-human primate (NHP) experiments. It includes eight experimental adjuvant groups, each incorporating an additional immunostimulatory molecule, alum, alongside the control group. As a note, all groups have 25 µg alum present. The table details the specific adjuvant compositions and their respective formulation types, illustrating the diverse approaches taken to enhance the immune response to the HPV vaccine.

Group	Immunostimulatory Molecules	Formulation Type	Abbreviation	References
1	Alum	Alum-absorbed suspension	Alum	[26,27]
2	Alum + Cationic Lipid	Lipid nanoparticle	Alum + LNP	[28,29]
3	Alum + Chitosan	Polymer nanoparticle	Alum + Chitosan	[30]
4	Alum + Cationic Lipid	Oil-in-water emulsion	Alum + OW	[3,29]
5	Alum + Squalene	Oil-in-water emulsion	Alum + Squalene	[31]
6	Alum + MPL + QS21	Liposome	Alum + MPL + QS21	[32,33]
7	Alum + MPL	Alum-absorbed suspension	Alum + MPL	[34,35]
8	Alum + Immune-stimulating complex (ISCOM)	Cage-like nanoparticle	Alum + ISCOM	[36]

## Data Availability

The data that support the findings of this study are available, but restrictions apply to the availability of these data, which were used under license for the current study, and so are not publicly available. Data are, however, available from the corresponding author upon reasonable request and with permission of Merck & Co., Inc. The underlying code for this study is not publicly available but may be made available to qualified researchers upon reasonable request from the corresponding author.
